# Development of an immune-related signature for predicting survival outcome and immunotherapy response in osteosarcoma

**DOI:** 10.18632/aging.203671

**Published:** 2021-11-08

**Authors:** Qinghua Wang, Wenjing Zhang, Yuxian Guo, Yuting Li, Kaifeng Fu

**Affiliations:** 1 Department of Health Statistics, Key Laboratory of Medicine and Health of Shandong Province, School of Public Health, Weifang Medical University, Weifang, Shandong 261053, China; 2 Tianjin Cancer Institute, National Clinical Research Center for Cancer, Key Laboratory of Cancer Prevention and Therapy of Tianjin, Tianjin Medical University Cancer Institute and Hospital, Tianjin 300060, China; 3 Department of Orthopedics, Sunshine Union Hospital, Weifang, Shandong 261061, China

**Keywords:** osteosarcoma, immune signature, prognosis, immunotherapy efficacy, biomarker

## Abstract

Osteosarcoma (OS) is the most common bone cancer, mainly diagnosed in children and adolescents. So far, no reliable molecular biomarkers have been identified to effectively evaluate OS prognosis and immune infiltration. Herein, we curated transcriptome profiles and clinical information from the publicly available OS cohorts to establish an immune-related prognostic signature. Besides, immunotherapeutic cohorts of urothelial cancer and melanoma patients were also employed to infer immunotherapy prediction roles of the identified signature. Lymphocytes infiltration, immune response-related pathways and signatures in the microenvironment were assessed according to distinct risk subgroups. Based on the univariate Cox analysis and further feature selection implemented by the LASSO regression model in the TARGET cohort, a 21-immune-gene signature was identified by combing the expression values and corresponding coefficients. We observed that the low-risk score of this signature was significantly linked with the preferable survival outcome (Log-rank test *P* < 0.001). The consistent results of better prognoses of the low-risk group were also obtained in subsequent two validation cohorts. Immunology analyses showed that favorable immune infiltration and elevated enrichment of immune response signals may contribute to the better outcome of the low-risk OS subgroup. The immunotherapeutic efficacy analyses demonstrated that low-risk patients harbored significantly enhanced response rates and improved immunotherapy survival outcomes. Together, our established signature could evaluate survival risk and represent the immune microenvironment status of OS, which promotes precision treatment and provides a potential biomarker for prognosis prediction and immunotherapy efficacy assessment.

## INTRODUCTION

Osteosarcoma is the most common malignant bone cancer and tends to be diagnosed in children and adolescents. Distal femur (43%), proximal tibia (23%), and humerus (10%) are the most common locations [1]. Significant pain and swelling of influenced bones are essential features, and OS could induce pathological characteristics in some situations. The overall survival rates are 67% after 2 years, 49% after 5 years, and 42% after 10 years [2]. It is noticeable that 15% to 20% of OS patients have metastasized at the diagnosis stage, and the survival outcomes of these patients are poor [2, 3]. Therefore, in addition to the current clinical and pathological methods for evaluating the cancer survival risk, novel and effective molecular biomarkers are urgently needed to promote individualized treatment for OS patients.

The therapy for osteosarcoma has advanced from amputation to limb-sparing surgery with implants [4]. Recently, the emergence of immunotherapies, such as adoptive cellular therapy, vaccination, and immune checkpoint blockade agents, has been becoming a promising treatment strategy [5, 6]. Several studies have demonstrated the encouraging findings of programmed cell death 1 (PD-1) and its ligand blockade therapy in both a humanized OS model and the lung metastases of OS [7, 8]. Nevertheless, in a randomized clinical trial conducted by Tawbi et al. [9], only 1 of 22 patients (5%) with OS responded to pembrolizumab, an anti-PD-1 agent. Recently multiple studies have emphasized the vital roles of tumor immune microenvironment on cancer tumorigenesis, progression, and treatment [10–12]. Indeed, immunotherapy efficacy depends on the anti-tumor immunocompetence of infiltration lymphocytes in the microenvironment [4]. The elevated activity of infiltration lymphocytes could induce the transformation of cancer cells from cold to hot by activating the immune system, which markedly enhanced tumor cytotoxicity and achieved high cure rates with minimal side effects [13]. However, there has been no suitable implement based on immune-related genes to effectively assess the tumor microenvironment and guide survival outcome or immunotherapeutic response of OS patients. Therefore, it is useful to construct a robust immune-relevant signature for evaluating the microenvironment status, survival risks, and treatment effects of OS patients.

In this study, we focused on establishing an immune signature with prognosis assessment and immune infiltration prediction based on the curated immune-related genes from the Immunology Database and Analysis Portal (ImmPort) database [14]. The transcriptomic expression profiles and clinical characteristics from the Therapeutically Applicable Research to Generate Effective Treatments (TARGET) project, Gene Expression Omnibus (GEO) database, and the Cancer Genome Atlas (TCGA) project were used for discovery and validation. We then calculated whether this signature was connected with prognosis and tried to figure out the association of the signature risk score with microenvironment-related indexes in OS. And finally, to elucidate the immunotherapeutic roles of the identified signature, we evaluated the abilities of this immune signature in determining immune responders from immune checkpoint inhibitors (ICI) treatment. Findings from this work would provide clues for guiding prognosis and tailoring immunotherapy strategies of OS patients.

## RESULTS

### Construction of immune-related risk signature in OS

**Figure 1:**
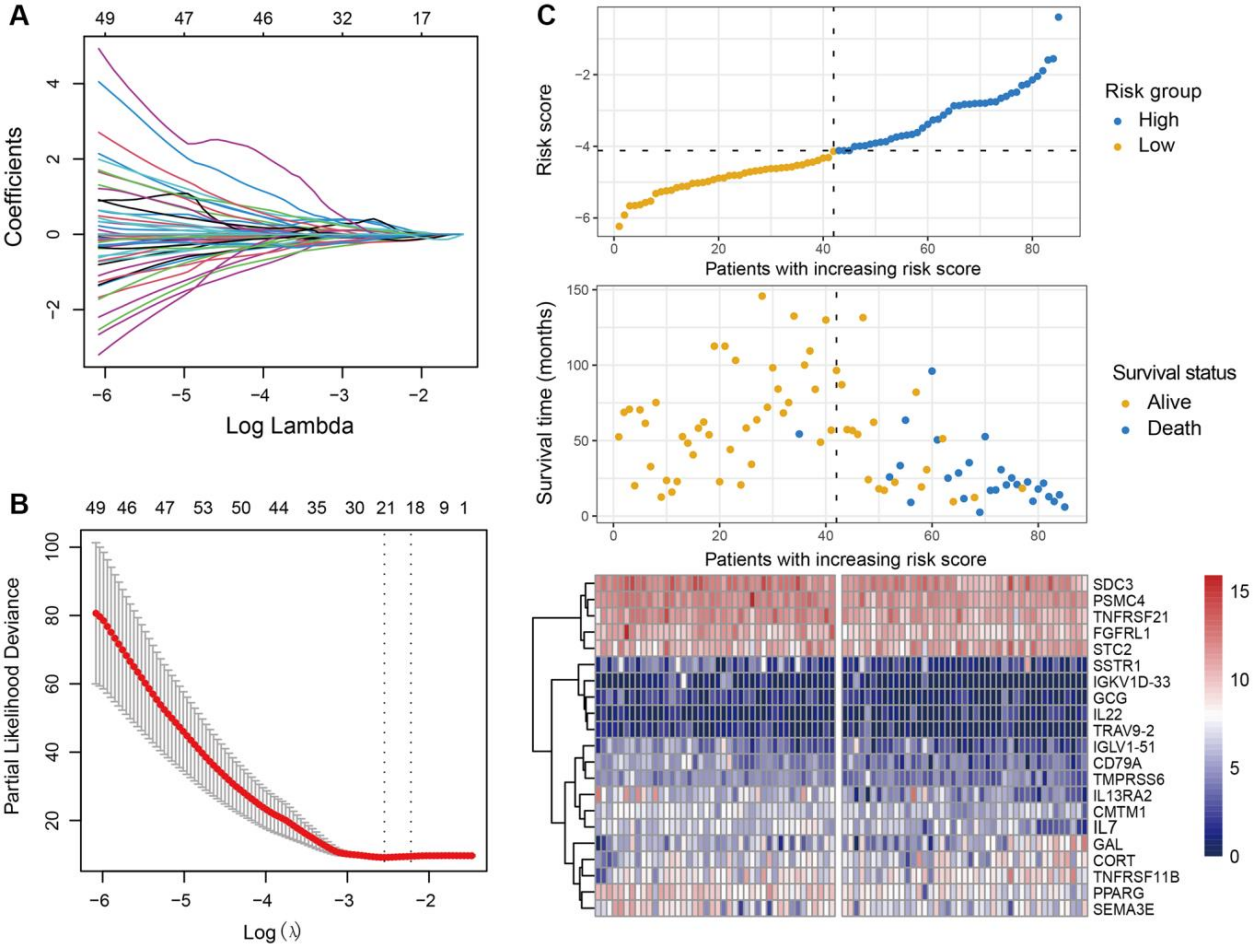
**Development of the immune-related risk signature.** (**A**) Regression coefficient profiles of the prognostic immune genes generated by the LASSO model in the TARGET cohort. (**B**) Partial likelihood deviance representation of distinct gene combination in LASSO regression. The red dots indicated the partial likelihood of deviance values, the gray lines indicated the standard error (SE), the two vertical lines on the left and right respectively indicated optimal values by minimum criteria and 1-SE criteria. (**C**) Heatmap of the 21 immune-related genes within the identified immune signature and the risk score curve based on the LASSO coefficients. OS patients were stratified into high-risk and low-risk subgroups with the median risk score.

Considering that the TARGET cohort is a special database for OS and contains the largest samples size. We therefore selected it as the discovery cohort to explore the immune-relevant signature that is linked with the survival risk of OS patients. The univariate Cox regression analysis was conducted in all of 1793 immune genes for the TARGET cohort, and a total of 328 prognostic genes were determined (all *P* < 0.05, Supplementary Table 1). The 328 immune prognostic genes were subsequently subjected to the LASSO regression model with 10-fold cross-validation setting to obtain a better regression model. The LASSO coefficient profile was generated against the log(k) sequence, and the minimize k method led to 21 optimal coefficients (Figure 1A and 1B). Finally, a developed model with 21 immune-related genes reached the optimal regression efficiency to speculate the prognostic ability.

The determined immune-relevant genes involved in antigen processing and presentation (PSMC4), cytokines (STC2, TNFRSF11B, CORT, GAL, CMTM1, IL7, GCG), cytokine receptors (SSTR1, TNFRSF21, FGFRL1, SDC3, IL13RA2), chemokines (SEMA3E), antimicrobials (IL22, TMPRSS6, PPARG), BCR signaling pathway (CD79A, IGLV1-51, IGKVID-33), and TCR signaling pathway (TRAV9-2). Moreover, we established an immune risk signature to assess the risk score of each patient by combing the 21 genes mRNA expression levels and their corresponding coefficients weighted by the LASSO method (Figure 1C, Supplementary Table 2). A heatmap of the determined 21 genes expression profile and the scatterplot of overall survival with corresponding risk scores for OS were shown in Figure 1C.

### The prognostic ability of the identified immune signature

**Figure 2:**
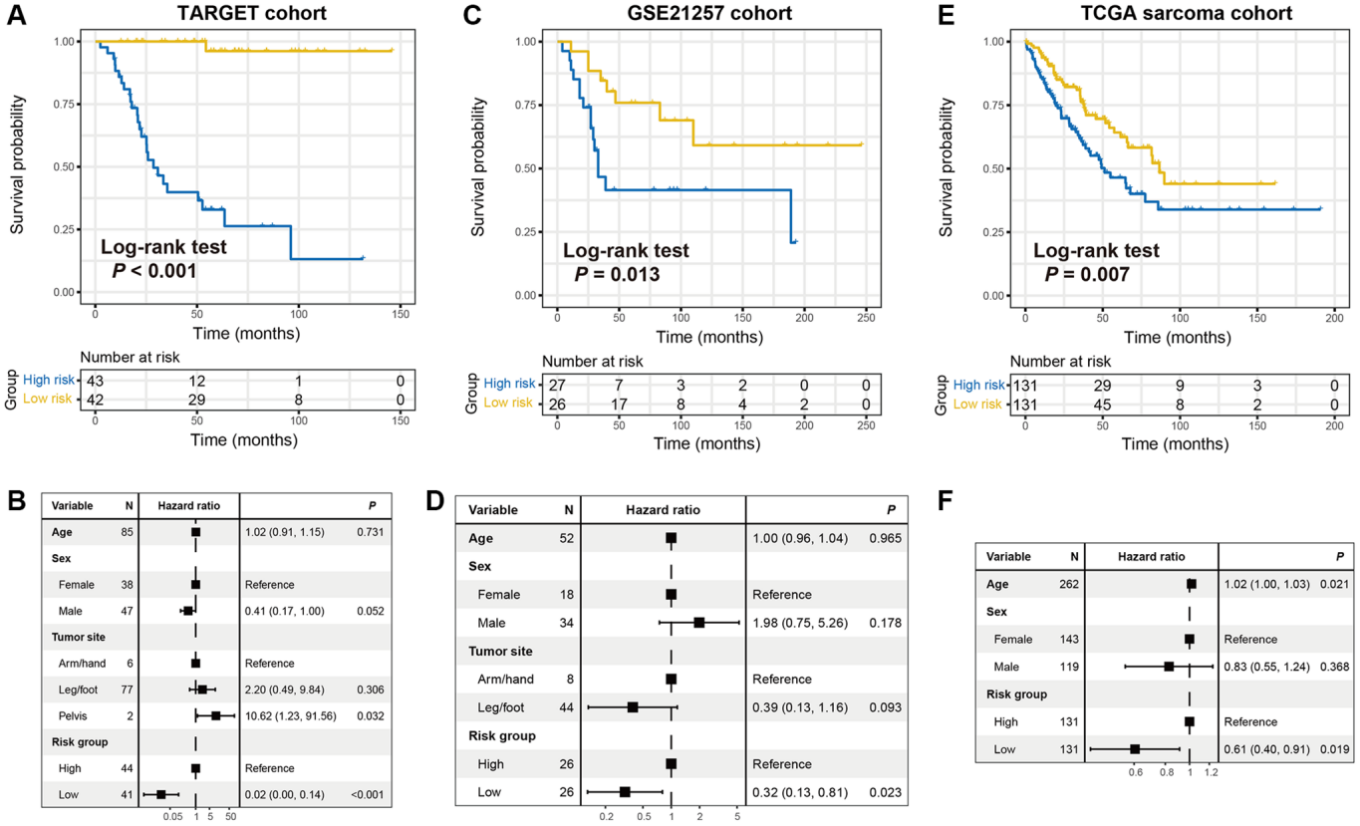
**The constructed immune risk signature was linked with OS prognosis.** Kaplan-Meier survival curves according to distinct immune signature subgroups in (**A**) the TARGET discovery cohort, (**C**) GSE21257 cohort, and (**E**) TCGA sarcoma cohort. (**B**, **D**, **F**) Forest plot representation of the multivariate Cox regression models delineated the connection between immune risk signature and prognosis in the three datasets. Clinical confounding factors were taken into consideration.

To explore the immune risk signature contribute to OS prognosis evaluation, we partitioned the samples of the TARGET cohort into a low-risk subgroup (*n* = 42) and a high-risk subgroup (*n* = 43) by applying the median risk score as the cut-off value. We observed that OS patients with the low-risk scores were significantly connected with a preferable prognosis as compared with those in the high-risk group (Log-rank test *P* < 0.001; Figure 2A). This connection remained still significant in the multivariate Cox model with age, sex, and tumor site taken into account (HR: 0.02, 95% CI: 0.00–0.14, *P* < 0.001; Figure 2B).

To confirm that the 21-gene-based immune signature classifier harbored a similar prognostic ability in distinct populations, we further validate this connection in the GSE21257 cohort with 53 OS samples. In the corroboration dataset, we also observed that patients of low-risk group exhibited a favorable survival outcome than those in the high-risk group (Log-rank test *P* = 0.013; Figure 2C). Multivariate Cox regression model further demonstrated that the immune-related signature could serve as an independent indicator for OS prognosis even adjusting for clinical confounding factors (HR: 0.32, 95% CI: 0.13–0.81, *P* = 0.023; Figure 2D).

We also curated 262 sarcoma samples from the TCGA cohort to further corroborate the prognostic value of the identified immune signature. Consistently, the significantly better survival outcome was observed in patients with low-risk scores under univariate Kaplan-Meier analysis (Log-rank test *P* = 0.007; Figure 2E) and multivariate Cox regression model (HR: 0.61, 95% CI: 0.40–0.91, *P* = 0.007; Figure 2F). ROC curves of the identified immune signature risk scores of the 3 cohorts used in this study (TARGET cohort area under curve [AUC]: 0.951; GSE21257 cohort AUC: 0.623; TCGA sarcoma cohort: 0.701) were illustrated in Supplementary Figure 1.

### The identified immune signature predictive of lymphocyte infiltration and immune response

**Figure 3:**
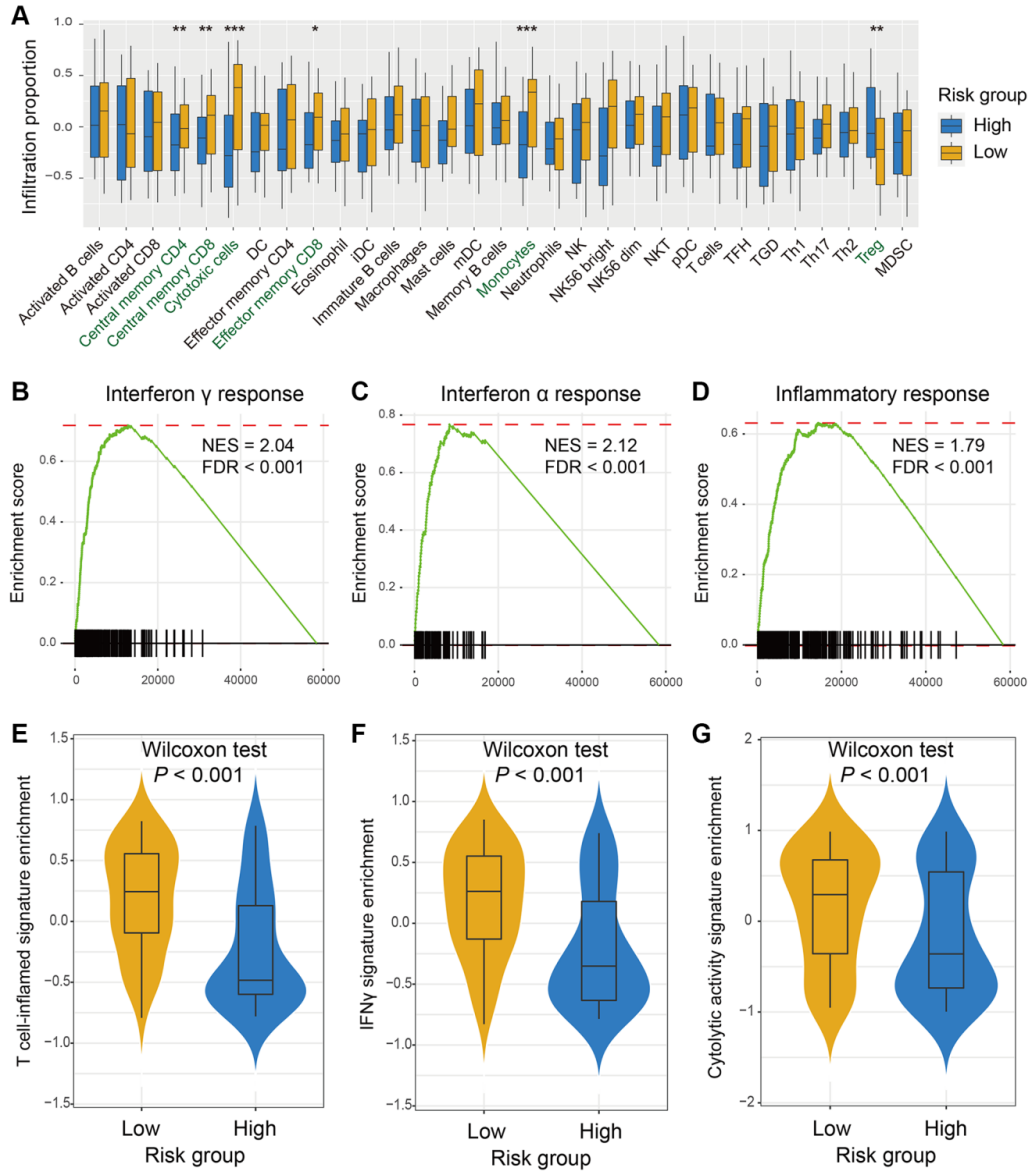
**The risk signature predictive of immune infiltration and immunotherapy response indicators.** (**A**) A total of 31 lymphocytes infiltration in low-risk versus high-risk subgroups was evaluated by the Angelova et al. algorithm. Pathways involved in immune response, such as (**B**) interferon γ response, (**C**) interferon α response, and (**D**) inflammatory response were markedly enriched in OS patients with low-risk signature scores. The elevated enrichment of immunotherapy efficacy predictors of (**E**) T cell-inflamed signature, (**F**) IFNγ signature, and (**G**) cytolytic activity signature were also connected with low-risk scores.

Since the risk signature was identified based on the immune-related genes, we hypothesized that its existence may be associated with lymphocyte infiltration, signaling pathways enrichment, and immune response. Therefore, we achieved a multiple-group boxplot by using ssGSEA to infer and visualize the relative infiltration levels of 31 immune cell subpopulations in low-risk versus high-risk subgroups from the TARGET cohort (Figure 3A). Anti-tumor lymphocyte subtypes, like central memory CD4^+^ and CD8^+^ T cells, effector memory CD8^+^ T cells, cytotoxic cells, and monocytes were markedly enriched in patients with the low-risk scores (all *P* < 0.05). However, the regulatory T cells (Treg), which belong to pro-tumor leukocytes, were exhibited decreased infiltration in the low-risk subgroup (*P* < 0.01). Furthermore, GSEA analysis against the OS gene expression data and Hallmark reference datasets demonstrated the biological signaling pathways associated with this immune signature. Genes involved in interferon γ response, interferon α response, and inflammatory response signaling pathways were remarkably enriched in patients from the low-risk group (Figure 3B–3D).

The three well-known signatures, including IFNγ response, T cell-inflamed GEP, and cytolytic activity signatures were identified to be associated with immunogenicity and immunotherapy response. Herein, we utilized these three signatures to investigate the association between the novel determined signature and immune response. In the TARGET cohort, the significantly elevated enrichment of T cell-inflamed signature (Figure 3E), IFNγ signature (Figure 3F), and cytolytic activity signature (Figure 3G) was noticed in the low-risk subgroup (Wilcoxon rank-sum test all *P* < 0.001). These findings suggested that OS patients with the reduced enrichment of this immune signature may be more responsive to immunotherapy.

### The immune signature for the prediction roles of immunotherapy efficacy

**Figure 4:**
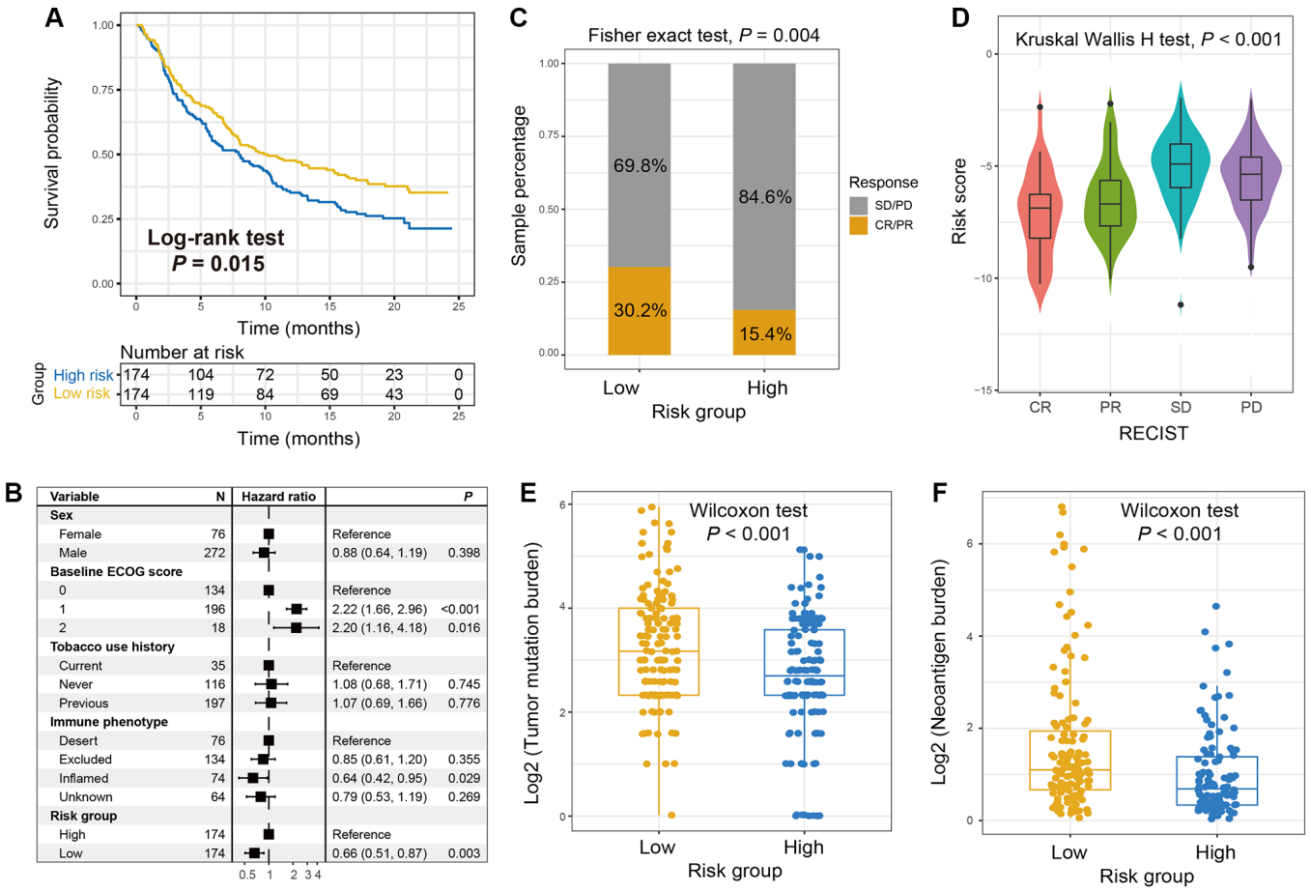
**The roles of the immune signature in evaluating ICI treatment efficacy for advanced urothelial cancer (UC).** (**A**) Kaplan-Meier survival plots of low-risk versus high-risk subgroups in the anti-PD-L1-treated UC cohort. (**B**) Forest plot of the multivariate Cox regression models with sex, ECOG score, smoking status, and immune phenotype taken into account to exhibit the association of the identified immune signature with ICI survival. (**C**) The proportion of therapeutic advantages to anti-PD-L1 therapy in low versus high-risk OS subgroups. (**D**) Distribution of immune signature risk scores in patients with distinct ICI treatment effect. (**E**) Tumor mutation burden and (**F**) neoantigen burden distribution in low-risk versus high-risk OS subgroups with the genomic data from UC immunotherapy cohort.

Immune checkpoint inhibitors (ICI) treatments represented by anti-PD-1/PD-L1 or anti-CTLA-4 agents have dramatically improved the survival outcome of advanced cancer patients. We therefore retrospectively acquired the transcriptomic expression profiles and clinicopathologic characteristics from an anti-PD-L1-treated cohort (Imvigor210) of advanced urothelial cancer (UC), so as to evaluate the link between the identified immune risk signature and immunotherapy efficacy. In this UC ICI cohort, patients with the low-risk scores harbored a significantly prolonged survival outcome as compared with those in the high-risk group (Log-rank test *P* = 0.015; Figure 4A). The link between low-risk signature scores and better ICI prognosis still existed in the multivariate Cox model with multiple clinical factors adjusted (HR: 0.66, 95% CI: 0.51–0.87, *P* = 0.003; Figure 4B). The significantly elevated response rate of ICI treatment was also detected in patients of low-risk subgroup (30.2% vs. 15.4%, Fisher exact test, *P* = 0.004, Figure 4C; Kruskal-Wallis H test, *P* < 0.001, Figure 4D). Further analysis demonstrated that the markedly enhanced tumor mutation burden (TMB) and neoantigen burden (NB) were both observed in OS patients with the low-risk scores, which are closely predictive of immunotherapy outcome (Wilcoxon rank-sum test both *P* < 0.001; Figure 4E and 4F).

**Figure 5:**
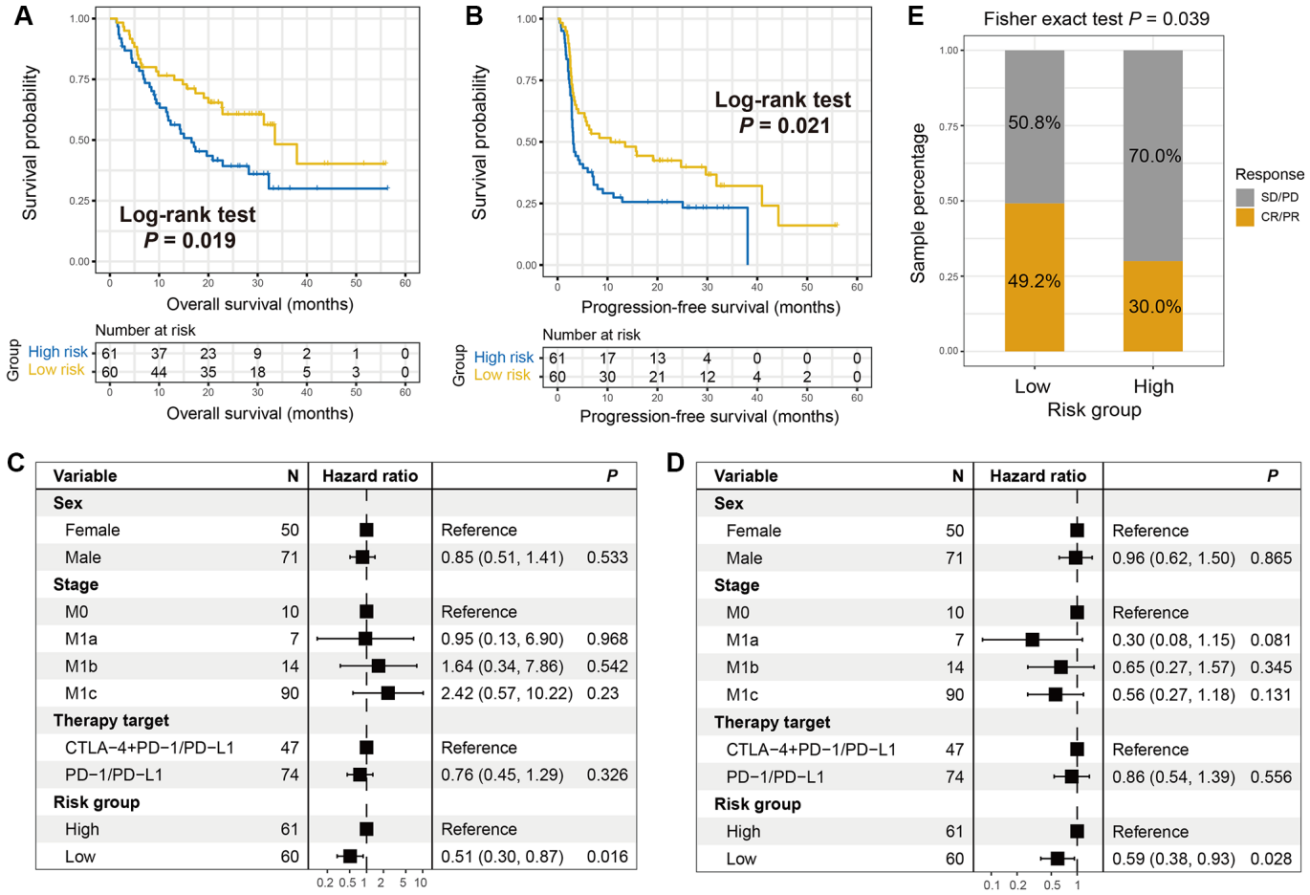
**The roles of the immune signature in evaluating ICI treatment efficacy for melanoma.** (**A**) Overall survival (OS) rate and (**B**) progression-free survival (PFS) rate differences of low- and high-risk groups were illustrated by Kaplan-Meier survival curves. Multivariate Cox regression models were conducted with sex, stage, and treatment type taken into account to elucidate the connections of the immune signature with (**C**) OS and (**D**) PFS outcomes. (**E**) The proportion of melanoma ICI responders in patients with low-risk versus high-risk signature scores was illustrated.

Another immunotherapeutic cohort of advanced/ metastatic melanoma patients (treated with anti-PD-1/ PD-L1 or anti-CTLA-4 agents) was also curated for validating the ICI predictive roles of the immune risk signature. In this melanoma population, we observed that patients in the low-risk group exhibited both significantly extended overall survival (OS) and progression-free survival (PFS) outcomes (Log-rank test *P* = 0.019 and 0.021, respectively; Figure 5A and 5B). Under multivariate-adjusted analysis, these connections remained still significant even controlling for sex, stage, and therapy type (OS: HR, 0.51, 95% CI, 0.30–0.87, *P* = 0.016, Figure 5C; PFS: HR, 0.59, 95% CI, 0.38–0.93, *P* = 0.028, Figure 5D). Conformably, the higher proportion of ICI-responsive melanoma patients was also noticed in the low-risk subgroup (49.2% vs. 30.0%, Fisher exact test *P* = 0.039; Figure 5E). The above results indicated that the immunotherapy efficacy may be evaluated by using the newly identified immune-related risk signature.

## DISCUSSION

It has long been known that the tumor immune microenvironment is a crucial mediator in tumor initiation and progression [15], however, these perspectives have not generated a propounding influence on routine clinical practice. This emphasizes the vital roles of the tumor microenvironment in predicting OS patients’ survival risk. In this research, we constructed a robust immune-related prognostic signature based on 21 immune genes in the discovery cohort and subsequently validated its efficacy in two independent datasets. The identified signature could divide OS patients into two subgroups represented distinct lymphocyte infiltration levels and immunotherapy response factors. Importantly, in the additional ICI-treated cohorts, this signature was demonstrated that its low-risk scores were markedly connected with the preferable ICI responses and outcomes. Therefore, this newly determined signature may serve as a potential surrogate for the survival prediction and immunotherapy efficacy evaluation of OS.

This study revealed that the immune risk signature was linked with OS patients’ survival outcome and this association still reached statistical significance even adjusting for clinical confounding variables. More importantly, our risk signature was obtained based on immune-relevant genes and demonstrated the connections with immunotherapy predictors of IFNγ response, T cell-inflamed signature, and cytolytic activity signature. Considering the important roles of these three signatures in predicting the clinical benefits to ICI treatments [16, 17], we hypothesized that OS patients with low-risk signature scores were more likely responsive to immunotherapy agents. Actually, in the ICI cohort of advanced urothelial cancer (UC), the low-risk scores were identified to connect with the elevated TMB and neoantigen burden, which were two well-known predictors of ICI efficacy. Nevertheless, the association of low-risk scores with high mutational and neoantigen burden was not observed in the melanoma ICI cohort (Supplementary Figure 2). Indeed, the better ICI outcomes of patients with low-risk signature scores were noticed in both UC and melanoma cohorts. These findings suggested that the established immune risk signature was contributed to identifying immunotherapy responders; however, the underlying mechanisms may not include the genomic mutational burden.

Recently, multiple studies with a focus on immunology characteristics have changed the orientations to the clinical tumor areas. Individual featured immune cells, such as CD4^+^ and CD8^+^ T cells have been demonstrated the prognosis prediction implications and drug treatment roles for patients diagnosed with OS by coupling with distinct signaling pathways and specific regulators [18–20]. Comprehensive knowledge has revealed the immunosuppression roles of regulatory T cells in multiple cancer types [21, 22]. The lymphocyte subtypes evaluation methods (e.g., CIBERSORT [23] and Angelova et al. method [24]) were frequently employed to characterize the immune infiltration landscape and calculate the connection with treatment effects. Our research leveraged the algorithm proposed by Angelova et al. and revealed that elevated central memory T cells (CD4 and CD8^+^), effector memory CD8^+^ T cells, cytotoxic cells, and reduced suppressive regulatory T cells infiltration were markedly detected in patients with low-risk scores. Moreover, signaling circuits with respect to the IFNγ and α responses, and inflammatory responses were markedly enriched in the low-risk subgroup, indicating that the identified immune risk signature was a superior predictive indicator for OS immune infiltration.

Recently several studies have also been established the immune-related signatures or discovered the prognosis-related immune genes in OS [25–27]. Wen et al. developed a tumor microenvironment-related risk model and employed the model score to stratify OS patients into two distinct subgroups. By using the differential analysis of the identified two OS groups, 3 genes (i.e., *COCH*, *MYOM2*, and *PDE1B*) were determined to construct the immune signature to evaluate the survival outcomes of OS patients [25]. Another study also developed an immune-related risk model based on 12 long non-coding RNAs and 3 immune genes. After dividing OS patients into high- and low-risk subpopulations, the authors observed that the low-risk subgroup exhibited a markedly improved prognosis and the preferable immune infiltration [26]. Similarly, Zhang et al. used the differential immune-relevant genes to construct a risk prognosis model and found that OS patients with low-risk scores harbored a better survival outcome and a favorable immune infiltration [27]. All these 3 studies provided stronger biomarkers for clinical OS prognosis prediction and treatment. Nevertheless, the lack of immunotherapy efficacy validation for the identified risk signatures or models may be a limitation. Our study also employed the immune-related genomic data to identify a risk signature, and importantly, we further verified its potential implications in survival evaluation and immunotherapy effect to guarantee clinical utilization.

However, there are several limitations in our research. First, transcriptome level analysis could not perfectly reflect the immune infiltration state. Second, the findings derived from this genomic association analysis lack *in vitro* experimental validation. Finally, owing to the lack of OS cohorts with gene expression profiles being tested by ICI agents, we are unable to corroborate the connection between the identified immune signature and the immunotherapeutic efficacy. Therefore, investigations based on our discoveries must be done carefully.

In summary, this study determined a novel immune-related risk signature that can not only evaluate the OS patients’ prognosis and immune infiltration degree but also predict the immunotherapeutic responsiveness. This novel signature can be clinically employed for the selection of OS patients with improved survival outcomes, personalized treatment based on the immune risk scores, and the enrollment of OS patients who are suitable to receive ICI agents. Nevertheless, prospective validation studies are needed to verify the significance of this immune signature.

## MATERIALS AND METHODS

### Collection of genomic data and clinical information

Gene expression profiles and clinical characteristics of 85 OS samples were retrospectively acquired from the TARGET project, which was defined as the discovery cohort. For the validations, 53 OS samples and 262 sarcoma samples respectively derived from the GSE21257 cohort and TCGA project were employed. The detailed clinical features and sequencing platforms were shown in Supplementary Table 3. Two immunotherapeutic cohorts with gene expression data were utilized to confirm the ICI roles of the identified immune signature. A total of 348 patients with advanced urothelial cancer in the IMvigor210 cohort [28], which was treated with atezolizumab (anti-PD-L1 agents), were collected; and their transcriptome data and clinical indexes were acquired from http://research-pub.gene.com/IMvigor210CoreBiologies. Another immunotherapy cohort contained 121 melanoma samples [29], which received anti-PD-1/PD-L1 or combination treatments, was also curated for further validating the risk signature response to ICI treatments. The detailed clinical characteristics of the two ICI cohorts were illustrated in Supplementary Table 4 for urothelial cancer and Supplementary Table 5 for melanoma. The list of comprehensive immune-related genes, containing a total of 1793 genes, was obtained from the ImmPort database [14] and shown in Supplementary Table 6.

### Construction of the immune signature by the LASSO regression model

Univariate Cox regression analysis was used to determine the prognostic immune genes based on the expression profiles of 1793 genes in the TARGET cohort. After obtaining the above prognosis-related genes, the LASSO regression model with 10-fold cross-validation (implemented by R glmnet package) was performed to identify the final genes mostly contributive to the survival model. By combing the identified specific gene expression values and corresponding coefficient, the immune risk signature was obtained. In the subsequent analyses, patients were stratified into the high-risk and low-risk subgroups with the median risk score of the immune signature as cut-off value.

### Quantify lymphocyte infiltration and immunotherapy response predictor enrichment

A study published by Angelova et al. proposed an 812-immune-metagene to conclude distinct tumor infiltration of 31 immune cell subtypes and depict a comprehensive immune landscape [24]. In this work, by applying the specific feature genes (Supplementary Table 7) that belong to distinct immune cells, we calculated the infiltration scores of each cell type for each sample. CIBERSORT is the most frequently used method to evaluate lymphocyte infiltration in cancer research [23]; nevertheless, in the discovery cohort (i.e., TARGET dataset), a majority of samples could not obtain a spendable estimation value. We therefore employed Angelova et al. algorithm to evaluate immune infiltration.

Recent several studies have demonstrated the crucial roles of microenvironment-based signals for assessing cancer immunotherapy efficacy. The IFNγ-related signature, which is a signal locates in the central site of the antitumor immune and predicts the clinical response to PD-1 blockade [16]. T cell-inflamed gene expression profiles (GEP) signature, which is consisted of 18 inflammatory regulators linked with immune response [16]. Cytolytic activity signature [17] is defined based on the transcriptomic expression levels of two vital cytolytic modulators, GZMA and PRF1, which are markedly upregulated upon CD8^+^ T cell activation [30] and during productive clinical responses to anti-CTLA-4 and anti-PD-L1 treatment [31, 32]. According to the representative genes of the above signatures (Supplementary Table 8), we inferred the enrichment scores for each sample and compared them in low-risk versus high-risk OS subgroups.

### GSEA and GSVA

Differential expression analysis of low- versus high-risk groups was conducted with the limma package [33]. The calculated t statistics of all genes were extracted and subsequently as input to perform gene set enrichment analysis (GSEA), which is embedded in the fgsea R package. Signaling pathways acquired from the Hallmark database were utilized as the annotation and reference circuits. The false discovery rate (FDR) and normalized enrichment score (NES) were calculated based on 1 million permutations. Single sample gene set enrichment analysis (ssGSEA) method within the GSVA package [34] was used to evaluate the enrichment scores of collected immune cells and signatures based on the specific feature gene panels.

### Definition of mutation burden and ICI responders in the immunotherapy cohorts

Tumor mutation burden (TMB) and neoantigen burden (NB) were defined as the log2 transformation of total mutation counts and neoantigen counts, respectively. For defining patients who responded to ICI treatments, patients with complete response (CR) or partial response (PR) were regarded to be efficacious to immunotherapy, and the rest were non-responders.

### Statistical analyses

R software (version 4.0.2) was utilized to perform all related calculations and plots. Gene expression profiles of identified 21 immune genes illustrated by heatmap were achieved with pheatmap R package. Kaplan-Meier method was applied to produce survival curves and the Log-rank test to compare the differences. Multivariate Cox regression model with clinical confounding factors taken into account was conducted under R forestmodel package. The relationship of two distinct groups with categorical variables was evaluated with Fisher exact test, while the continuous variable was Wilcoxon rank-sum test. Association between multiple subgroups and continuous variables was calculated with the Kruskal-Wallis H test. Two-sided *P* values less than 0.05 were considered to be statistically significant unless a particular specification.

### Data availability

All data used in this study are acquired from publicly available OS cohorts.

## Supplementary Materials

Supplementary Figures

Supplementary Table 1

Supplementary Tables 2 and 3

Supplementary Tables 4-8
