# Supplementary material for: Development of an immune-related signature for predicting survival outcome and immunotherapy response in osteosarcoma
Source: Aging (Albany NY). 2021 Nov 8;13(21):24155–70. doi: 10.18632/aging.203671 (PMC8610143; doi:10.18632/aging.203671)
Supplement: Supplementary Figures [file aging-13-203671-s001.pdf]

SUPPLEMENTARY FIGURES

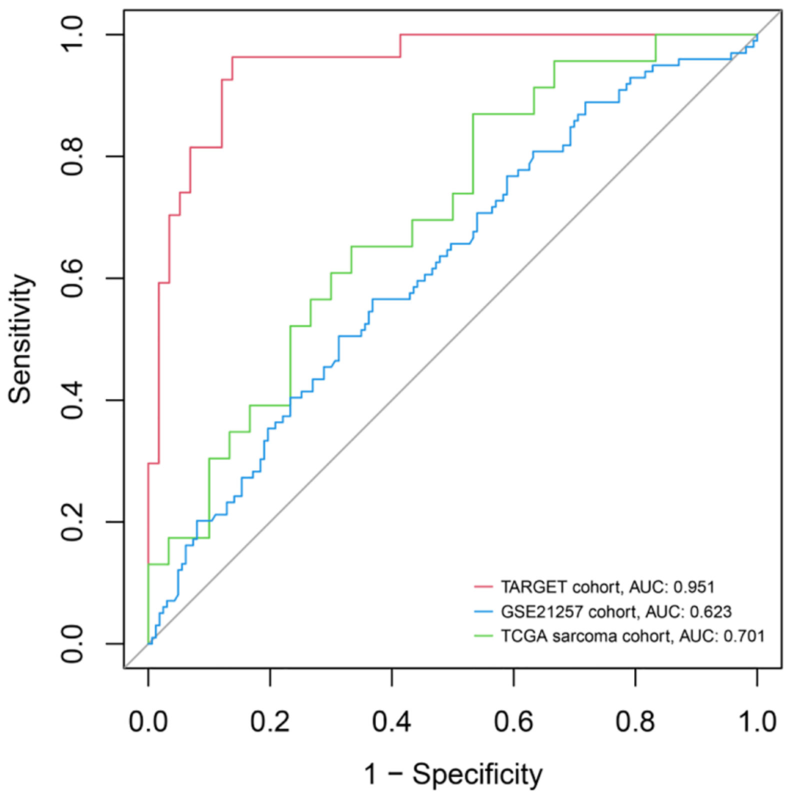

Supplementary Figure 1. ROC curves illustration of the identified immune signature risk scores of the 3 cohorts included in this study.

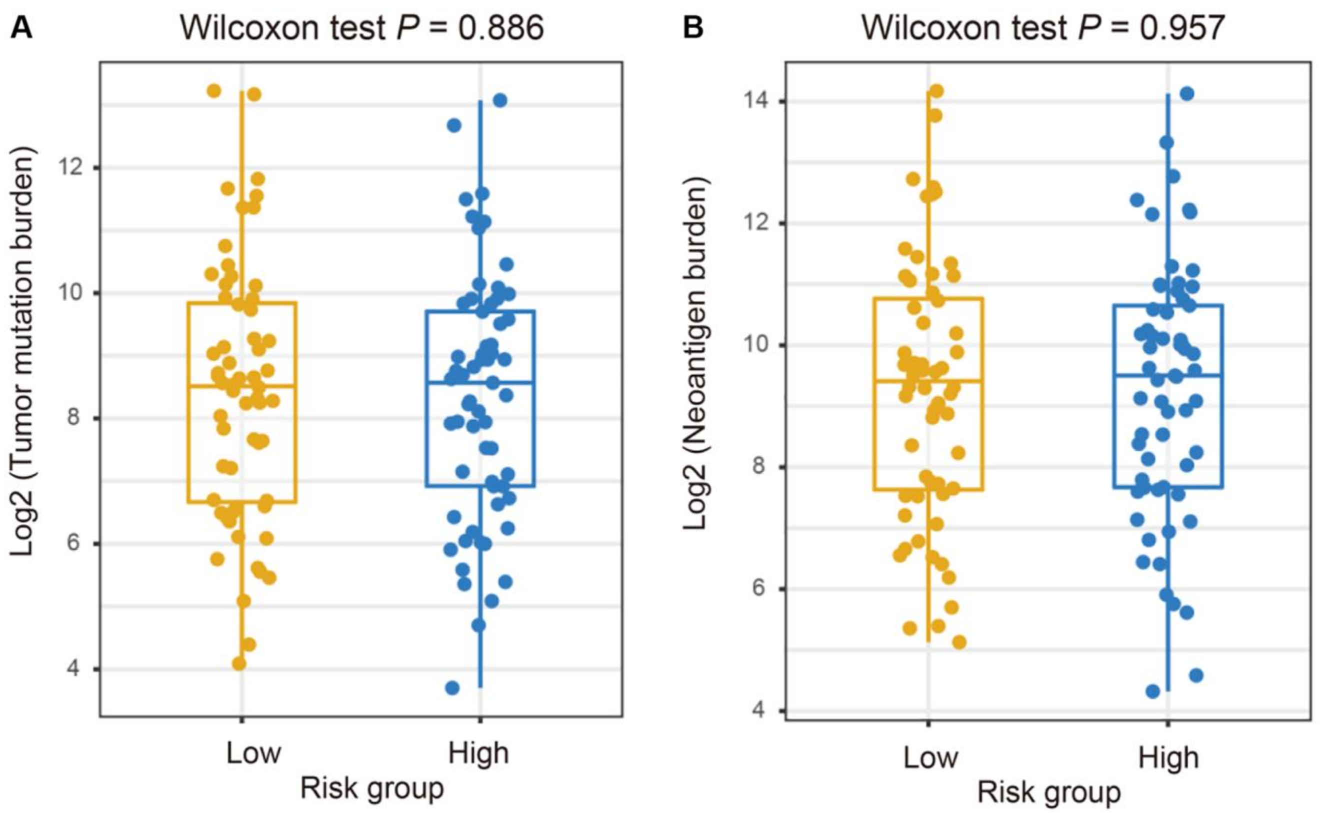

Supplementary Figure 2. The distribution of (**A**) TMB and (**B**) neoantigen burden in low-risk versus high-risk subgroups in the melanoma immunotherapy cohort.
