# Supplementary material for: Development of an immune-related signature for predicting survival outcome and immunotherapy response in osteosarcoma
Source: Aging (Albany NY). 2021 Nov 8;13(21):24155–70. doi: 10.18632/aging.203671 (PMC8610143; doi:10.18632/aging.203671)
Supplement: Supplementary Tables 2 and 3 [file aging-13-203671-s003.pdf]

SUPPLEMENTARY TABLES

Supplementary Table 2. The extracted immune-related genes and corresponding coefficients from LASSO regression model in the TARGET cohort.

Gene	Coefficient
STC2	0.141522237
TNFRSF11B	0.098754475
CORT	0.086711381
IL22	0.084649801
GAL	0.069818606
PSMC4	−0.013177704
CD79A	−0.014491145
SEMA3E	−0.017349815
IGLV1-51	−0.021744664
CMTM1	−0.0494132
SSTR1	−0.054281399
TNFRSF21	−0.057043444
TMPRSS6	−0.065932207
FGFRL1	−0.074868654
IGKV1D-33	−0.075750338
IL7	−0.078764237
TRAV9-2	−0.110319916
SDC3	−0.118469971
IL13RA2	−0.125900341
GCG	−0.183788709
PPARG	−0.205915021

Supplementary Table 3. Summary of three osteosarcoma datasets included in this study.

Datasets	Platforms	Sample size
TARGET	Human Exon ST Array (Affymetrix)	85
GSE21257	Illumina human-6 v2.0 expression beadchip	53
TCGA	Illumina RNAseq HTSeq	262
Total		400
